# Challenges of Assessment and Intervention for the Mental Health Issues in Adolescence and Young Adults

**DOI:** 10.1002/pchj.70032

**Published:** 2025-06-29

**Authors:** Raymond C. K. Chan, Emma Barkus, Yiqun Gan

**Affiliations:** ^1^ Neuropsychology and Applied Cognitive Neuroscience Laboratory, State Key Laboratory of Cognitive Science and Mental Health, Institute of Psychology Chinese Academy of Sciences Beijing China; ^2^ Department of Psychology University of Chinese Academy of Sciences Beijing China; ^3^ Department of Psychology Northumbria University Newcastle Upon Tyne UK; ^4^ School of Psychological and Cognitive Sciences and Beijing Key Laboratory of Behavior and Mental Health and Key Laboratory of Machine Perception (Ministry of Education) Peking University Beijing China

Mental health is one of the main challenges in the 21st century. It is especially important for adolescents and young people. Maintaining positive mental health is critical during this period as people navigate developmental milestones to transition to adulthood. This period marks profound identity exploration, academic pressures, social integration, relationship development, and future planning. In an era marked by intense pressures to succeed, social dynamics, and rapid technological advancements, young people's mental health and well‐being have are a paramount concern. Traditionally, the adolescence stage has been defined as 10–19 years of age. However, recent literature has challenged this and extended it to 10–24 years (Sawyer et al. 2018). This means that promoting positive mental health over these years would involve engaging multiple agencies from child and adult healthcare providers, schools, universities, work related settings and families alike. This special issue focuses on the challenges of assessment and intervention for mental health concerns in adolescents and young adults. The body of work is being brought together to address the urgent need for research on the uniqueness of context and delivery of young people's mental health.

The collected work in the special issue reflects the cutting‐edge approach to assessment and intervention of mental health issues in this population. It will be published in two independent volumes. The current volume comprises 9 studies concerning anxiety, depression and anhedonia using different methodologies. Four studies adopted advanced statistical modeling and ecological momentary assessment to examine the relationship between anxiety, depression and stress. Sabah et al. (2025) found that online and offline social sensitivity could mediate the influence of online vigilance on anxiety, depression and stress among Algerian female college students. On the other hand, Guo and Li (2025) showed that an authentic inner compass is particularly important for subjective well‐being in among young Chinese adults who are anxiously attached, and that resilience explains the association between an inner compass and well‐being. Adopting an ecological momentary assessment, Feng et al. (2025) further demonstrated that in college students daily perceived stress could influence depression directly, or indirectly through rumination. Kuang et al. (2025) designed a scale specifically examining people‐pleasing behaviors in Chinese adults, and, reported that people who were high people pleases had lowest mental well‐being.

Two studies specifically examined the impact of COVID‐19 on college students' mental health based on a follow‐up design. Ni et al. (2025) showed that olfactory function affected depressive symptoms indirectly through chemosensory pleasure, and such mediation effect persisted over 3 months. Adopting cross‐lagged panel network analysis, Liu et al. (2025) findings further highlighted the strong relationships between anxiety and depression during the lockdown period and lockdown lift period in Chinese college students.

Three studies investigated the association between mental health and specific traits. Hu et al. (2025) adopted network analysis to examine the relationship between motivation and pleasure domain associated with social function. They showed that the motivation and pleasure domain accounted for the highest variance proportion of social functioning in college students. Moreover, people with high social anhedonia demonstrated a different network structure compared to people with low social anhedonia. Using FaceReader analysis, Zhang et al. (2025) further showed that there were different moderating effects of facial emotion expressions on people with autistic traits, schizotypal traits and social anhedonia. Wang et al. (2025) examined the relationship between glutamate levels of the anterior cingulate cortex and sensory integration in people with high and low social anhedonia. Their findings showed that higher glutamate levels at the anterior cingulate cortex were correlated with poor sensory integration in people with high social anhedonia.

These exciting and relevant findings collectively highlight the importance of continued research with people across the adolescence and young adult range. Although increasingly college samples are being dismissed in mental health research, the circumstances for individuals attending higher education reflect a potentially distinct set of circumstances and stressors such that they need additional consideration for effects on mental health and mental well‐being. With widening participation as a policy for higher education sectors internationally, the diversity of young people attending university settings is increasing. Adolescence continues to be important to consider as a unique interaction of social, emotional, and biological factors. Although substantial gains have occurred in mental health research for these ages to date, there are still more questions to address. The research considered here highlights novel methodological or statistical methods as well as demonstrating the importance of considering individual differences in adolescents and young adults.

In this Special Issue, the collection of published articles has showcased a variety of advanced methodologies. Many studies have employed sophisticated statistical techniques such as ecological momentary assessment and cross‐lagged panel network analysis, whereas others have delved into the realm of neuroscience. However, most of the contributions still rely predominantly on self‐report measures. Although self‐report is undoubtedly a crucial component, the future of adolescent mental health assessment lies in the integration of more objective methods. These include biomarkers such as cortisol, biochemical indicators, and neuroendocrine approaches. Moreover, with the advent of the digital age, incorporating machine learning, large language models, and natural language processing into mental health assessment holds great promise. We anticipate that future Special Issues will specifically focus on and explore these cutting‐edge technologies, particularly how they can be applied to the mental health assessment of adolescents. The integration of multi‐modal approaches, including leveraging the latest advancements in large language models, is particularly exciting and will undoubtedly enhance our understanding and evaluation of adolescent mental health.

## Conflicts of Interest

The authors declare no conflicts of interest.

## Data Availability

Research data are not shared.
